# Obituary

**Published:** 1889-01

**Authors:** 


### Obituary.

Died at his home in Alameda, California, Dr. C. C. Knowles, on Monday, December 17th, 1888, in his 70th year. He was a native of New Hampshire. In early life he moved to Lowell, Massachusetts, and studied dentistry with Dr. Ambrose Lawrence, a practitioner well known in his profession. Dr. Knowles removed to Whitesboro, New York, where, in 1842, he married Mrs. K. E. Slade, of Lowell, and after practicing for a time at Whitesboro, he returned to Lowell and established a large and lucrative practice. The gold fever of California so attracted him that in 1852 he removed to the Pacific Coast. He was one of the first dentists to establish a practice in San Francisco. At that time dentistry was a crude science compared with that of the present day. Dr. Knowles was a man of much active and acquired ability, and was thorough in his work, and was soon at the head of his profession in that part of the country. In 1874 Dr. Knowles came east to attend a meeting of the American Dental Association, on that trip he had a sun stroke from the effects of which he never fully recovered. In 1877 his failing health compelled him to give up his practice, which passed into the hands of his sons, Drs. W. A. L. and S. E. Knowles. In 1878 his first wife having died some years before, he married Mrs. Mary Carson. In 1879 he took up his residence in Alameda and remained there till the time of his death. During his prime Dr. Knowles was a very active and prominent man in public affairs. In 1870 he was the chief mover in organizing the California State Dental Association, he was its first President, and was re-elected several subsequent terms. He was for many years a member of the Board of Education in San Francisco, and for a number of years its President. Dr. Knowles was a man of sterling integrity, who always did what he conceived to be right. He was most kind and genial in disposition, and the news of his death will bring a cloud of sadness to all who knew him, and the sympathy of all such will be extended to the bereaved ones of his family.

